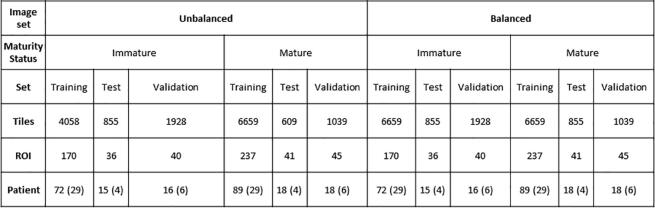# HistoClean: Open-source software for histological image pre-processing and augmentation to improve development of robust convolutional neural networks

**DOI:** 10.1016/j.csbj.2021.08.033

**Published:** 2021-08-26

**Authors:** Kris D. McCombe, Stephanie G. Craig, Amélie Viratham Pulsawatdi, Javier I. Quezada-Marín, Matthew Hagan, Simon Rajendran, Matthew P. Humphries, Victoria Bingham, Manuel Salto-Tellez, Richard Gault, Jacqueline A. James

**Affiliations:** a Patrick G Johnston Centre for Cancer Research, Queen’s University Belfast, Belfast, Northern Ireland; b Belfast Health and Social Care Trust, Belfast, Northern Ireland; c The School of Electronics, Electrical Engineering and Computer Science, Queen’s University Belfast, Belfast, Northern Ireland; d The Institute of Cancer Research, London United Kingdom

**Keywords:** Digital image analysis, Image pre-processing, Image augmentation, Artificial intelligence, Open-source software, HistoClean, AI, Artificial Intelligence, DIA, Digital Image Analysis, GUI, Graphical User Interface, ROC, Receiver-Operator Characteristic, AUC, Area Under Curve

## Abstract

The growth of digital pathology over the past decade has opened new research pathways and insights in cancer prediction and prognosis. In particular, there has been a surge in deep learning and computer vision techniques to analyse digital images. Common practice in this area is to use image pre-processing and augmentation to prevent bias and overfitting, creating a more robust deep learning model. This generally requires consultation of documentation for multiple coding libraries, as well as trial and error to ensure that the techniques used on the images are appropriate. Herein we introduce HistoClean; a user-friendly, graphical user interface that brings together multiple image processing modules into one easy to use toolkit.

HistoClean is an application that aims to help bridge the knowledge gap between pathologists, biomedical scientists and computer scientists by providing transparent image augmentation and pre-processing techniques which can be applied without prior coding knowledge.

In this study, we utilise HistoClean to pre-process images for a simple convolutional neural network used to detect stromal maturity, improving the accuracy of the model at a tile, region of interest, and patient level. This study demonstrates how HistoClean can be used to improve a standard deep learning workflow via classical image augmentation and pre-processing techniques, even with a relatively simple convolutional neural network architecture. HistoClean is free and open-source and can be downloaded from the Github repository here: https://github.com/HistoCleanQUB/HistoClean.

## Graphical abstract

**Figure:**
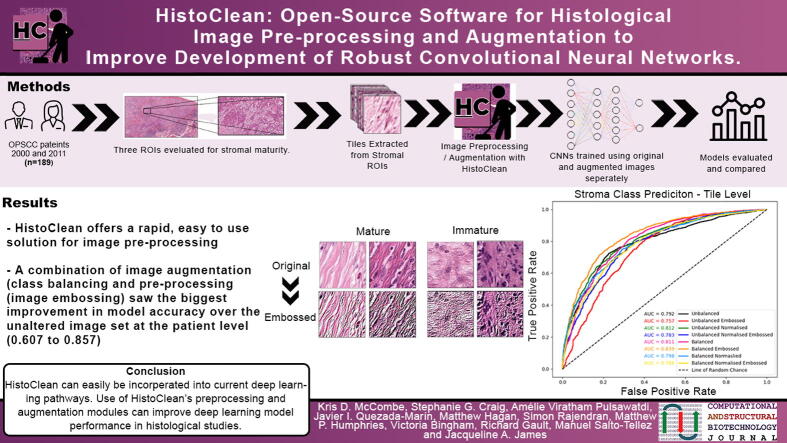

## 1 Introduction

The growth of digital image analysis in clinical pathology and its subsequent case for use in clinical medicine has been supported by the conception of open-source digital image analysis (DIA) software [1], [2], [3]. Use of machine learning from predetermined features allows for the development of DIA algorithms within these software environments. This allows bio-image analysts and consultant histopathologists to answer difficult, specific research questions in human tissue [4]. The subsequent introduction of deep learning has revolutionised the development of DIA algorithms [5]. This has enabled potential solutions to tumour and biomarker detection, as well as tumour subtyping [6], [7]. However, these solutions require domain-specific knowledge relating to the deep learning methodology, as well as the awareness of hardware acceleration [8].

**Fig. 1:**
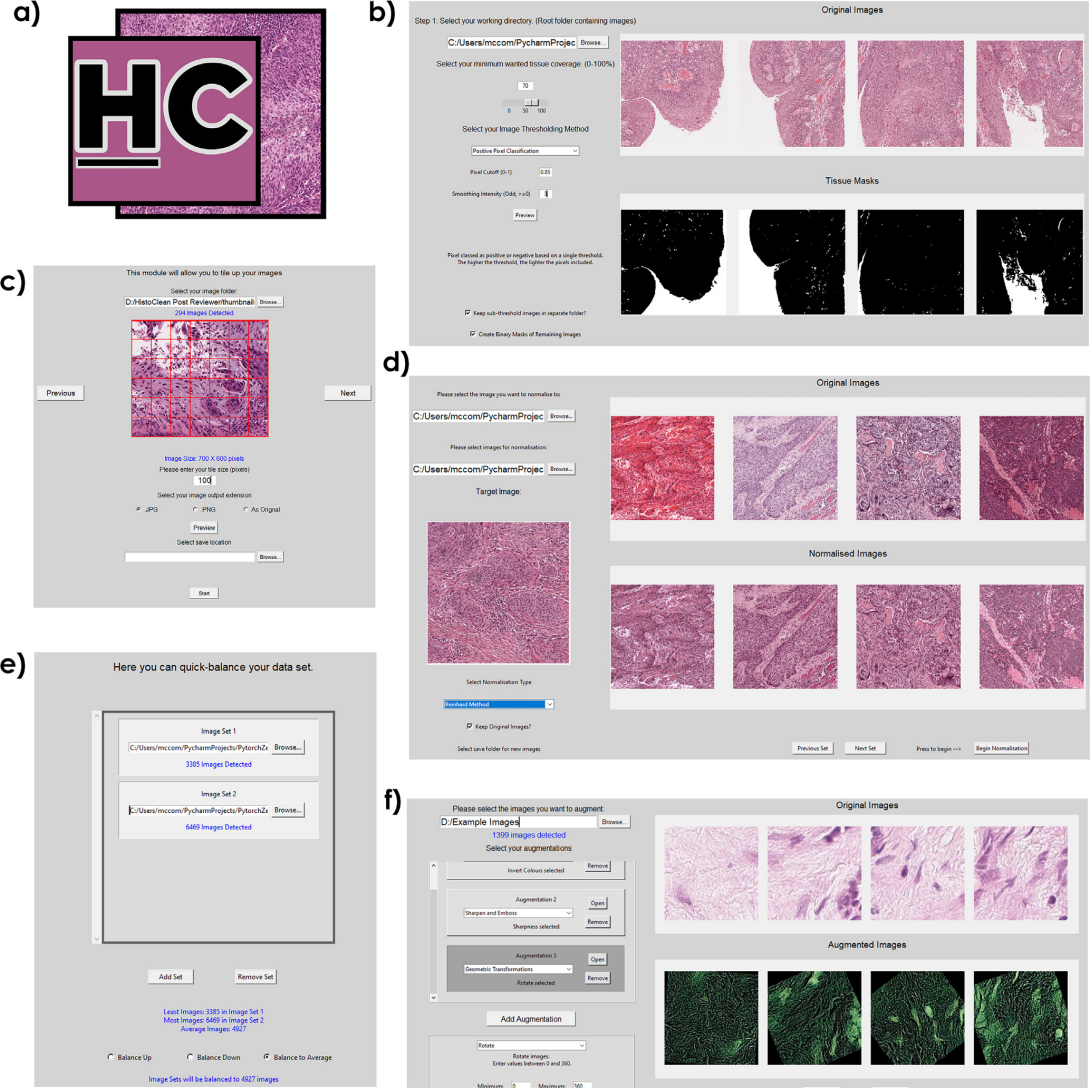
HistoClean (a), an all-in-one toolkit for the pre-processing of images for use in deep learning. Modules include (b) whitespace estimation and filtering, implemented in the white space removal module, (c) tools for generation of image tiles from larger images, which are executed within the image patching module. (d) Image normalisation, which standardises the colour grading of the images. (e) Quick balancing, which balances the number of images in different classes by classic image augmentation, and (f) image pre-processing/ augmentation, which provides further methods to expand an image set, add noise and accentuate image data.

Consequently, open-source software to aid bio-image analysts without a background in computer vision to develop deep learning models have evolved [9], [10]. Deep learning methodologies learn feature representations from the data without requiring predefined feature extraction. The resultant models can therefore be significantly more sensitive to dataset specific attributes, such as irregularities in staining, batch effects and the quality of the digital slide [11], [12]. Use of image pre-processing and augmentation prior to developing deep learning models can regularise the input images, thereby, mitigating the potential for bias in the training of the CNN, or other deep learning models, and its independent validation [13], [14], [15], [16]. Among these, the most common techniques include class-balancing [17], image normalisation [18], and image augmentation [19]. These techniques often involve the use of multiple coding libraries, which in turn requires knowledge of the documentation before implementation. Herein we present HistoClean; an open-source, high-level, graphical user interface (GUI) for image pre-processing. HistoClean aims to complement other open-source software and deep-learning frameworks in the bio-image analysis ecosystem [9], [10], [20]. HistoClean’s image pre-processing toolkit is divided into five functional modules based on computational methods frequently used in histological image pre-processing; image patching, whitespace thresholding, dataset balancing, image normalisation and image augmentation (Fig. 1). These modules can be used independently or in combination with each other as the user requires. HistoClean brings together image pre-processing techniques from across multiple Python libraries. This simplifies the image preparation phase of deep-learning analysis in a way that is transparent and maintains data integrity.

The process of developing deep learning models for histopathological analysis is a combined effort between computer scientists, biomedical scientists and pathologists. HistoClean aims to help bridge the knowledge gap between these domains by providing a point-and-click alternative to computer programming for these processes. The intended audience of this application are i) Biomedical scientists and pathologists, who can use the tool to evaluate how image pre-processing might influence visualisation of underlying biology. ii) Computer scientists who can apply the appropriate changes in a rapid and reproduceable way, saving the time and effort of developing coding scripts in the process.

**Fig. 2:**
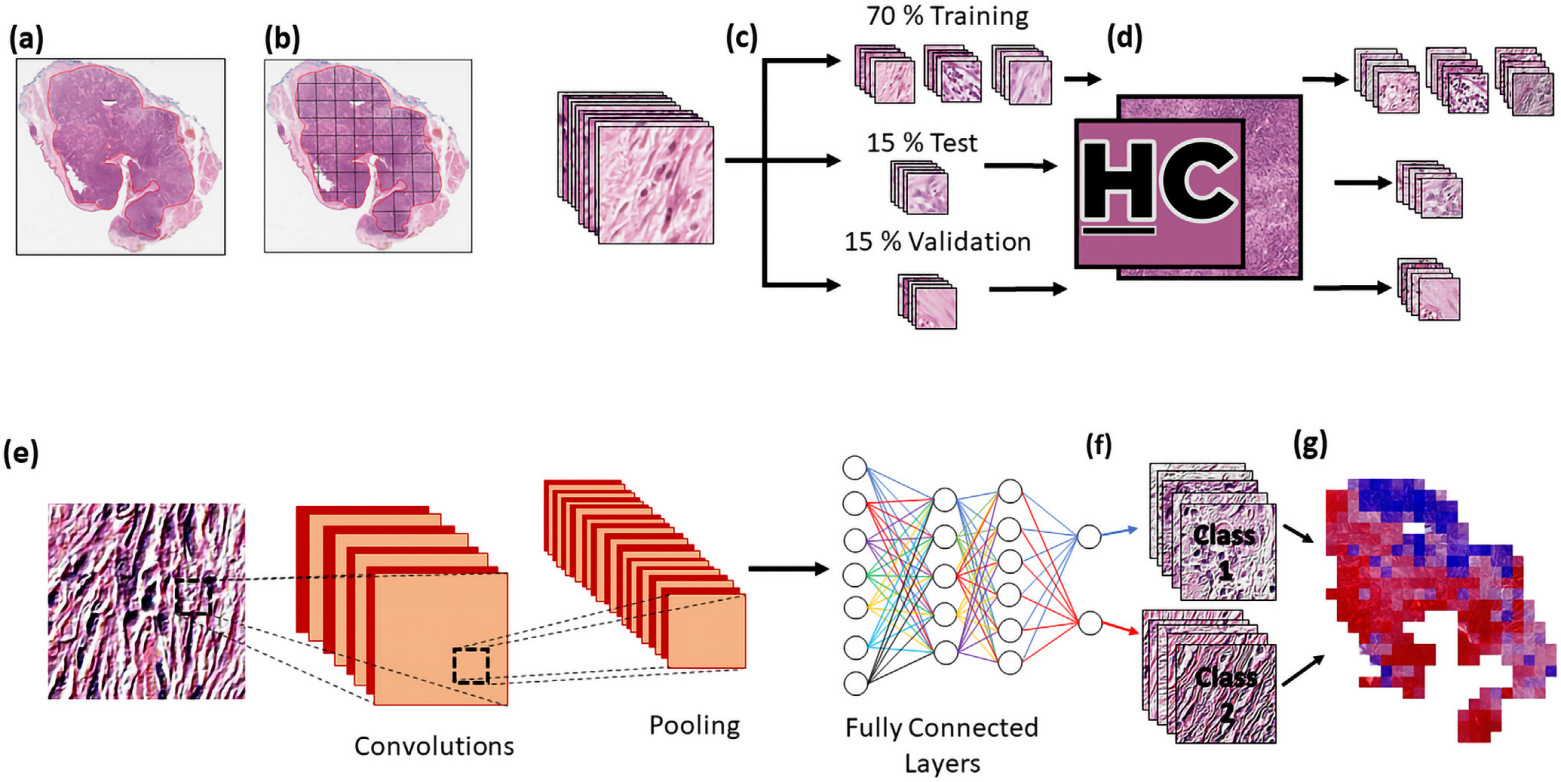
Use of HistoClean in the development of histology based convolutional neural networks. Slides are scanned at high-resolution, normally 20-40x (0.025 µm/px) magnification and are virtually annotated (as outlined in red) by a pathologist on a digital platform (a). Tiles of equal size are extracted from the virtual annotations (b). These tiles are independently sorted into training, test and validation datasets at a patient level (c). Image pre-processing and augmentation is conducted on the tiles using HistoClean where appropriate in the training, test and validation datasets in order to prepare tiles for use in a convolutional neural network (d). Within a typical convolutional neural network, each tile is fed through a series of convolutional and pooling layers in order to create feature maps to differentiate between the two classes (e). These feature maps are then fed through several fully connected layers which determine which class the images belong to (f). Each tile is assigned a value used for class prediction; the prediction values for each tile are then aggregated in order to provide an overall class prediction per patient (g). (For interpretation of the references to colour in this figure legend, the reader is referred to the web version of this article.)

In this study, a practical example of how HistoClean can optimise input images for training a simple CNN to predict stromal maturity is described (Fig. 2). In evaluating these models, we demonstrate the benefit of image pre-processing for deep learning, even in relatively simple CNN architecture, and introduce HistoClean as an open-source software solution to quickly implement and review these techniques.

The main contribution of this paper is the development of a novel, easy to use, point-and click application for the rapid pre-processing and augmentation of image datasets for use in deep learning, image analysis pipelines.

## 2 Materials and methods

### 2.1 HistoClean application development

HistoClean was developed using Anaconda3 and Python 3.8. Code was written using the PyCharm integrated developer environment. The GUI was developed using the Tkinter toolbox (v8.6). Initial development and testing of the software was performed on an Octane V laptop with an Intel Core i7-9700F 3.0 GHz processor and 32 GB Corsair 2400 MHz SODIMM DDR4 RAM, with a Windows 10 operating system. The application was converted to a .exe program using the Pyinstaller Python package [21]. All testing was performed in the Windows 10 operating system. For ease of use it is recommended that images should be organised within directories corresponding to each image class. The application runs all processes on the CPU. No GPU is required. The application makes prominent use of multithreading, which scales to the number of cores in the CPU. The application has 160 user interaction points, all of which have exception handling for input characters and data types. The application is designed to allow the user to have complete control over the techniques applied. The modules outlined here can be used together or separately as the user requires.

#### 2.1.1 User interface design

The HistoClean user interface was created utilising established simple-design principle, minimising the amount of on-screen text and interaction points while maintaining functionality [22]. The interface features a modular, single-window design with a focus on minimalism and displays clear categorisation of the application’s functions [23]. Icons were added to the module selection buttons to allow for quicker and easier identification of module functionality [24]. Upon selecting a module, users are walked through the process using the concept of procedural instruction [25] with a natural progression from the top of the screen to the bottom. We enhanced the principles of clarity and comprehensibility, with reduced focus on aesthetics [26]. The primary colouration of black on light grey/white was chosen not only for visual clarity, but for accessibility for colour-blindness. A wayfinding feature has been implemented into the module selection buttons, which darken according to which module is active at the time.

HistoClean features extensive error handling which follows the principles of prevention, correction and recovery [27]. Examples of how each of these principles is utilised is as follows: HistoClean will *prevent* the user from entering non-numeric values if these are not appropriate. HistoClean will also automatically *correct* for one-channel images in the Normalisation module by converting to RGB beforehand. Finally, throughout the entirety of the program, user-interaction points that have been accidentally overlooked can be *recovered* via the use of feedback tools such as popups and widget highlighting.

HistoClean is designed to be a standalone application. As such, the application was compiled as an executable file using Pyinstaller. All dependencies are included at download, with the user only needing to click on the application to begin.

#### 2.1.2 Image patching module

CNN’s require input image tiles to have consistent dimensions [28]. For this reason, HistoClean includes an image patching module that utilises the Python library *Patchify* [29]. This module interface allows the user to create image tile subsets from a larger input image to their specification and provides real-time feedback of the output to the user, facilitating straightforward evaluation and adjustment (Fig. 1a). This module can be used for block processing of *n* images organised within a common file directory. The user can select an output destination wherein the directory structure and naming conventions of the original images will be retained and populated with the requested image patches. The file names of these new image tiles are suffixed with their patch co-ordinates from the original image for reproducibility. Maintaining transparency in the pre-processing stages ensures that results can ultimately be traced back to their source ensuring that HistoClean does not damage original source data or impede data integrity and reproducibility.

#### 2.1.3 Tissue thresholding module

**Fig. 3:**
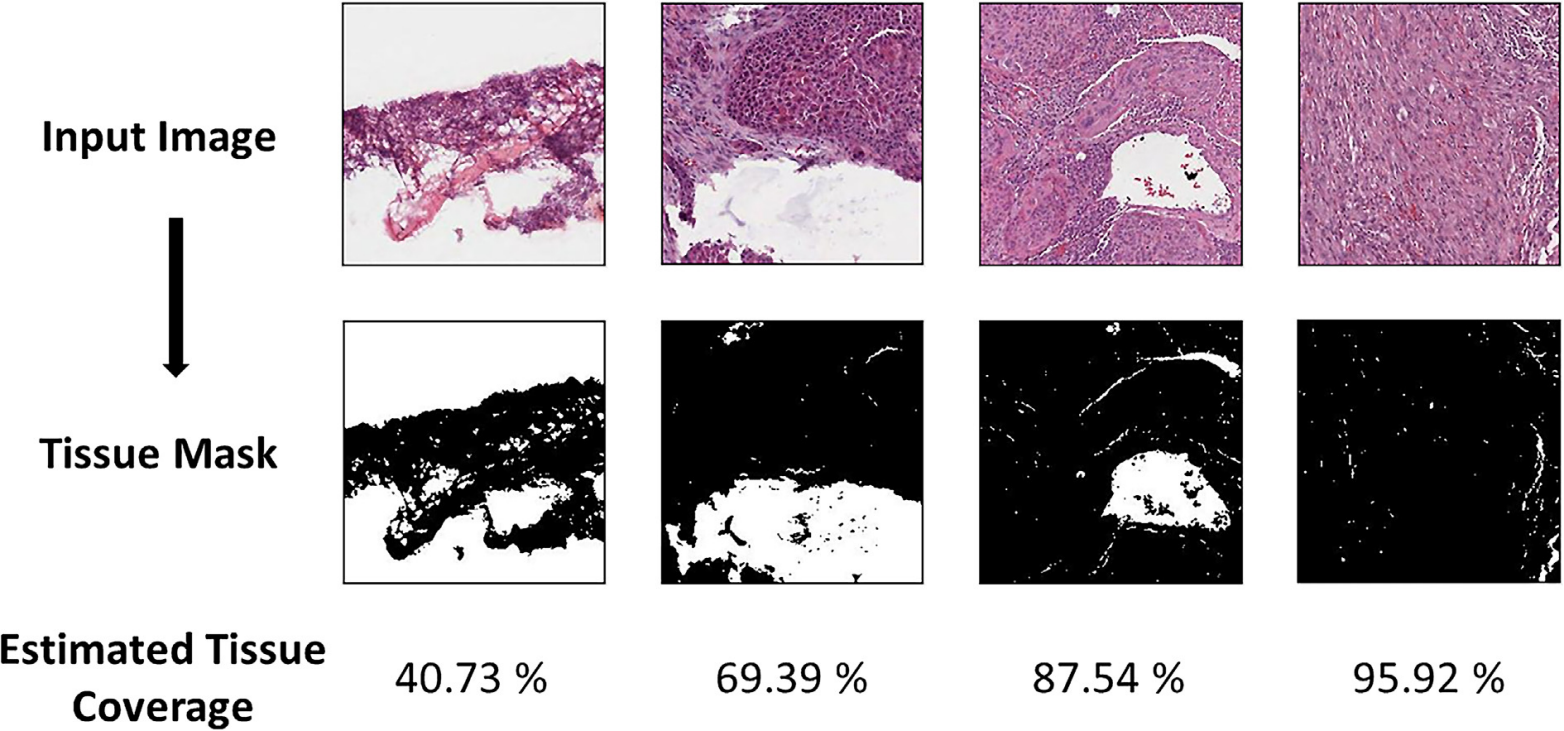
Four representative images demonstrating use of the positive pixel classifier method to estimate tissue coverage. All images were given the same cut-off (0.8). The top row contains the original images, with the bottom row showing the binary mask for tissue (black = tissue, white = whitespace). The bottom row shows the estimated tissue coverage within the image tile.

Most pathology-orientated CNN’s are developed to address questions within the tissue, therefore, an excess of whitespace in the input images may impair model development [30]. In order to address this issue and improve the quality of input image tiles, HistoClean includes a tissue thresholding module that allows the user to remove image tiles from their dataset based on a minimum threshold of approximate tissue coverage. The method outlined in this paper uses binary thresholding to determine the percentage of positive pixels, representing tissue, and null pixels, representing whitespace (Fig. 3). Tissue coverage and relative intensity of the staining can vary significantly depending on any number of predisposing factors. Therefore, HistoClean’s module interface allows the user, in real time, to explore different thresholds for dichotomising these pixels into tissue vs whitespace. In addition, adaptive thresholding is available for each image as well as Otsu binarization [31]. All of these thresholding options come courtesy of the OpenCV Python library [32]. These processes generate a binary mask for each image which the GUI presents alongside the original image for review. Users can view five images simultaneously. Upon approval of an arbitrary threshold, images are removed or relocated based on user preference.

#### 2.1.4 HistoClean: Class balancing module

Class balancing is essential to prevent class bias of data when developing deep learning models [33]. For this reason, HistoClean includes a class balancing module that enables the user to equalise the number of images per class prior to training of the CNN (Fig. 1c). This requires that each class of images be provided in a separate directory by the user. The user can then decide to balance using three options: reducing the number of image tiles in each class to the smallest class, increasing the number of image tiles in each class based on the largest class, or balance the number of images in each class based on the average number of images in each class. The pre-requisite for using this functionality is that no class contains less than one eighth of the samples of the largest class. This pre-condition is reinforced through exception handling. This is to prevent duplicate images arising from repeated augmentations. If the user balances the samples through class reduction, the image tiles in the larger class-specific dataset are then relocated to a new directory, denoted as ‘Removed Images’, or are permanently deleted based on user preference. If class-size is balanced by the addition of image tiles, then a random assortment of image tiles equal to the difference between the largest class-specific image dataset are selected without replacement from within the smaller dataset(s). The random selections of image tiles are then augmented thus balancing the number of image tiles in that class by addition of ‘new’ image data. Image augmentation techniques are randomly selected from mirroring, clockwise rotation at 90°, 180° or 270°, or a combination of mirroring and a single rotation. This can create up to 7 unique images from a single image as required. A random number generator, seeded to the date and time of dataset balancing, determines the augmentation applied.

#### 2.1.5 Image normalisation module

**Fig. 4:**
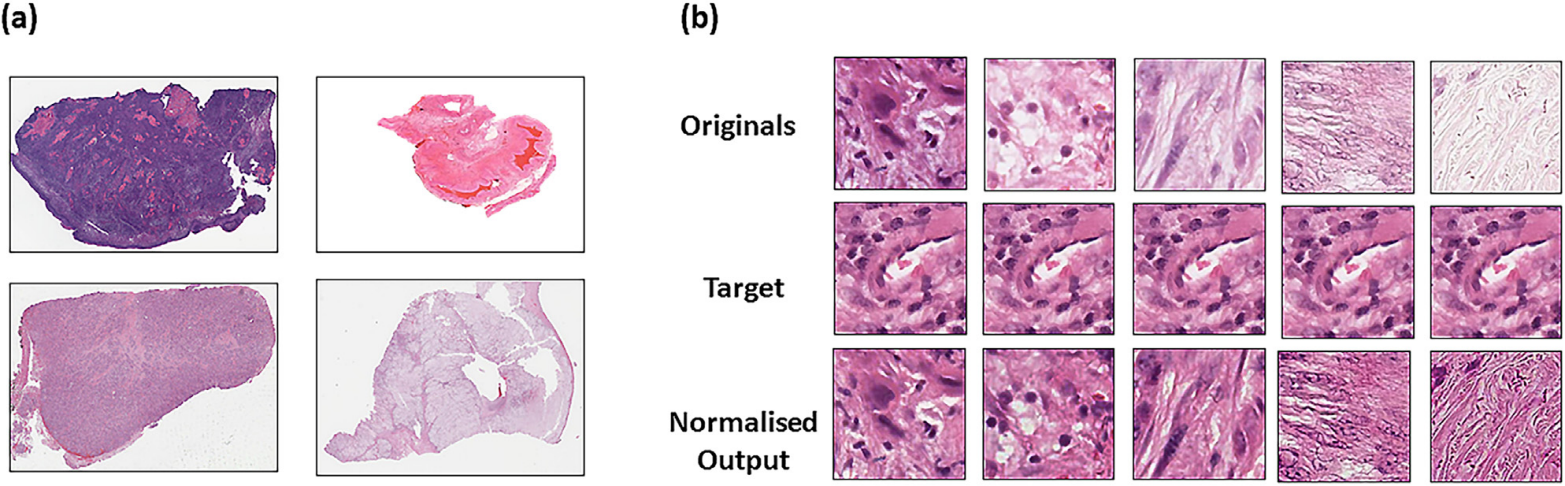
Image normalisation in histological images. Batch effects in haematoxylin and eosin staining and different staining protocols often leads to an inconsistent colour range in histological images as demonstrated by images taken from the TCGA head and neck diagnostic dataset (a). Demonstration of histogram normalisation to correct for the inconsistent colour range between samples while preserving histological architecture (b). The top row shows a selection of original un-normalised tiles, the middle row shows the target image and preferred colour range being normalised to and the bottom row shows the result of that normalisation.

Histological images possess unique image colour, contrasts, and brightness profiles. Batch effects in staining (Fig. 4a) can significantly influence model performance [13]. Image normalisation can be used to bring uniformity to the images in the dataset by adjusting the range of pixel values of an input image, according to that of a target image [18]. For this reason, HistoClean includes an image normalisation module based on histogram matching from the Python library *scikit-image* [34]. Histogram matching works by comparing the cumulative histogram of pixel intensities from a target and an input image, before adjusting the pixel values of the input image according to the target image [35] (Fig. 4b). HistoClean’s module interface allows the user to select a target image to normalise to and to review examples of the histogram-matched images before committing to image normalisation to *n* images organised within a folder. This gives the user complete control over the normalisation process. These can be either be for tiles for a singular slide or a cohort of slides. These are saved to a separate user-defined folder, or can replace the original images at the user’s discretion. If saved in a separate folder, the directory structure of the original is replicated.

#### 2.1.6 Image augmentation and Pre-processing module

It is not always possible to source large collections of histological images in the pursuit of developing deep learning models [36]. Image augmentation is a technique which that can be used for the artificial expansion of image datasets to provide more training examples. In addition, image pre-processing can be used to enhance features already present in an image dataset in order to provide more specific features for the CNN training [37]. By providing deep learning models with augmented data, the user can reduce the risk of overfitting and improve the generalisation ability of the CNN [36]. For this reason, HistoClean includes an image augmentation/pre-processing module based on the Python library *Imgaug* [38]. This allows the user to select, review and apply the most popular image augmentation techniques used in the development of CNNs to their image dataset in real-time (Fig. 1e). These include adjusting the colour range, contrast, blur and sharpness, noise, pixel and channel dropout and more.

There are over 50 pre-processing options available that can be used individually or in combination. Generated images files from augmentation are identifiable by their name, which incorporates the name of the root file from which the image derived so as to maintain data integrity. If a new image set is created, the directory structure is replicated from the original.

### 2.2 Patient samples

Ethical approval and access to diagnostic H&E stained slides from a retrospective cohort of oropharyngeal squamous cell carcinomas (OPSCC) for stromal maturity prediction by artificial intelligence was granted via the Northern Ireland Biobank (OREC 16/NI/0030; NIB19/0312) [39]. Briefly, patients with a primary oropharyngeal cancer diagnosed between 2000 and 2011 were identified and their diagnostic H&E retrieved from the Belfast Health and Social Care Trust courtesy of the Northern Ireland Biobank. All slides were digitised using a Leica Aperio AT2 at 40x magnification (0.25 μm / pixel). Virtual slides were saved in a .svs file format and imported into the open-source image analysis tool QuPath (v0.1.2) [1] to enable image annotation by a qualified histopathologist.

### 2.3 Classification of stromal maturity

**Fig. 5:**
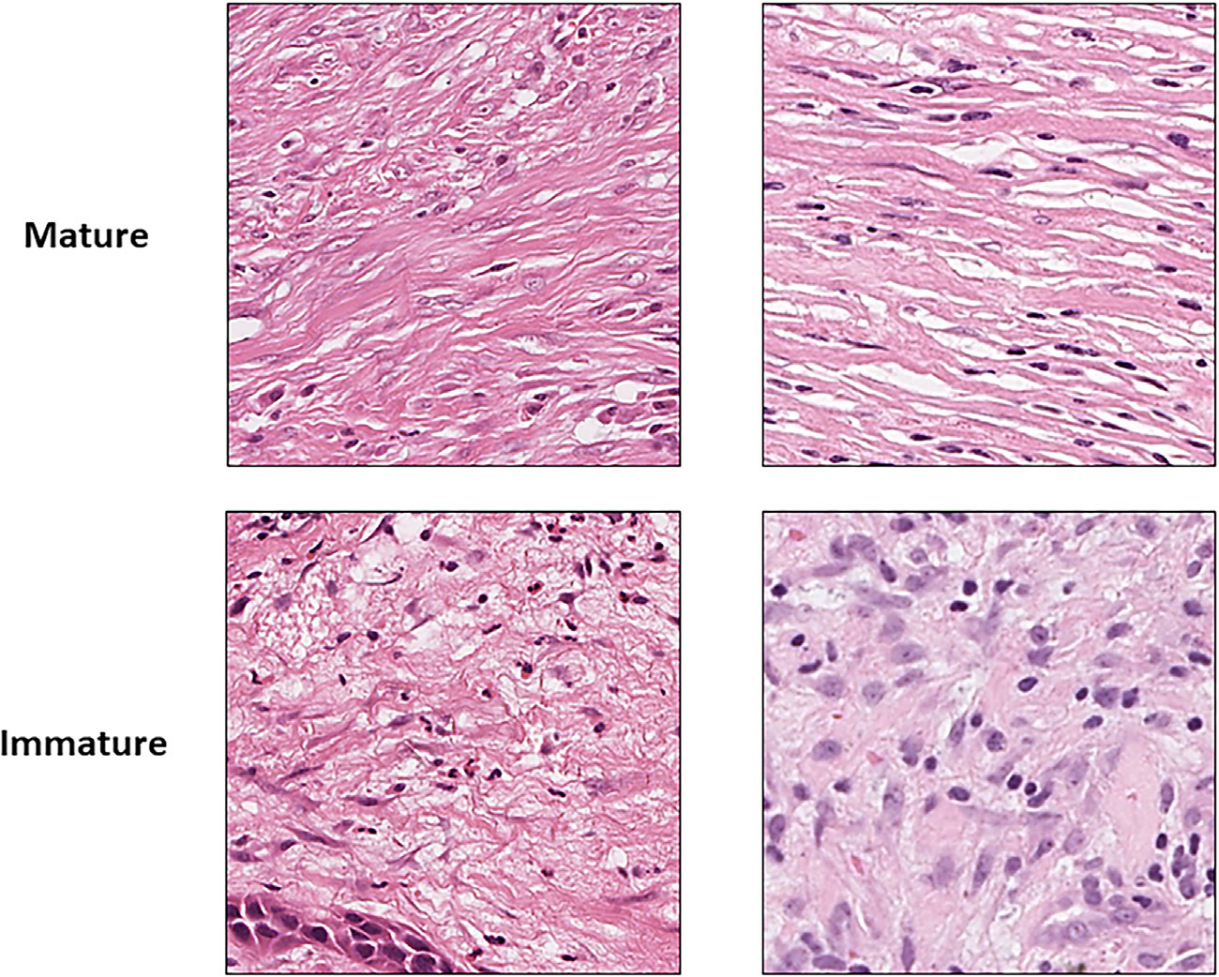
Reference images for mature (top row) and immature (bottom row) stroma randomly selected from the dataset. Images taken at 40x magnification and used as reference criteria during the manual classification of stromal maturity by the independent assessors in the study.

Using DIA software QuPath (v0.1.2), a trained pathologist reviewed all the diagnostic H&E slides from each case before identifying and annotating ROIs for classification of stromal maturity on the slide that most represented malignant OPSCC. QuPath was used due to the presence of the built-in tools available for the annotation of the ROIs. Classification of mature stroma was defined by the presence of fine, regular, elongated collagen fibres organised with approximately parallel orientation. Conversely, immature stroma was defined by disorganised, random orientation of collagen fibres with and without the presence of oedema and myxoid-like degeneration [40], [41]. Stroma maturity was determined as being either mature or immature for each ROI by visual review. This was conducted by the pathologist, along with two other blinded independent assessors based on previously published criteria [40], [41]. Stromal maturity is a prognostic factor in cancer, with immature stoma associated tumour patients exhibiting significantly worse survival. The exact mechanisms behind why this is the case are not fully understood, but theories have emerged citing stromal gene expression and the influence the desmoplastic reaction has epithelial to mesenchymal transition [42], [43]. Representative images of mature and immature stroma were created and used as reference criteria for all assessors prior to classification (Fig. 5).

### 2.4 Image set preparation

Image tiles of size 250X250 pixels at x40 (0.025 µm/pixel) magnification were extracted from the ROIs, that had been previously annotated in QuPath by the pathologist, using the built-in scripting functions. These dimensions and resolution were chosen to be large enough to allow the images to capture the intricacies of the stromal structure, but small enough to reduce computational expense and allow for larger training batch sizes. Tiles were organised in separate directories for mature and immature stroma as determined by manual assessment. These were further grouped into directories representing each patient. Images were divided at a patient level into three sets. First, the training set, which consisted of 70% of the patients was used to train the CNNs. Second, the test set, which consisted of 15% of the patients was used to evaluate model performance during training. Lastly, the independent validation set consisting of the remaining 15% of patients. This did not influence the training of the model and was instead used to evaluate model performance. This produced the baseline “Unbalanced” image set. Images were organised in this way to account for intra-patient heterogeneity of stromal maturity. An entire heterogenous patient existed within the training, test or independent validation set and was not split among the three. This is to prevent the CNN from “recognising” patients between the three sets.

### 2.5 Image pre-processing using HistoClean

In order to demonstrate the benefit of image pre-processing for the development of robust CNN’s, seven independent image datasets were produced from the baseline image set. These utilised a combination of class balancing, image normalisation and pre-processing (Table 1).

**Table 1:** Summary table of HistoClean modules used in each dataset. Columns denoted with an “X” show which modules were used.

Dataset	Balancing	Normalisation	Augmentation
Unbalanced			
Unbalanced Normalised		X	
Unbalanced Embossed			X
Unbalanced Normalised		X	X
Embossed			
Balanced	X		
Balanced Normalised	X	X	
Balanced Embossed	X		X
Balanced Normalised Embossed	X	X	X

**Fig. 6:**
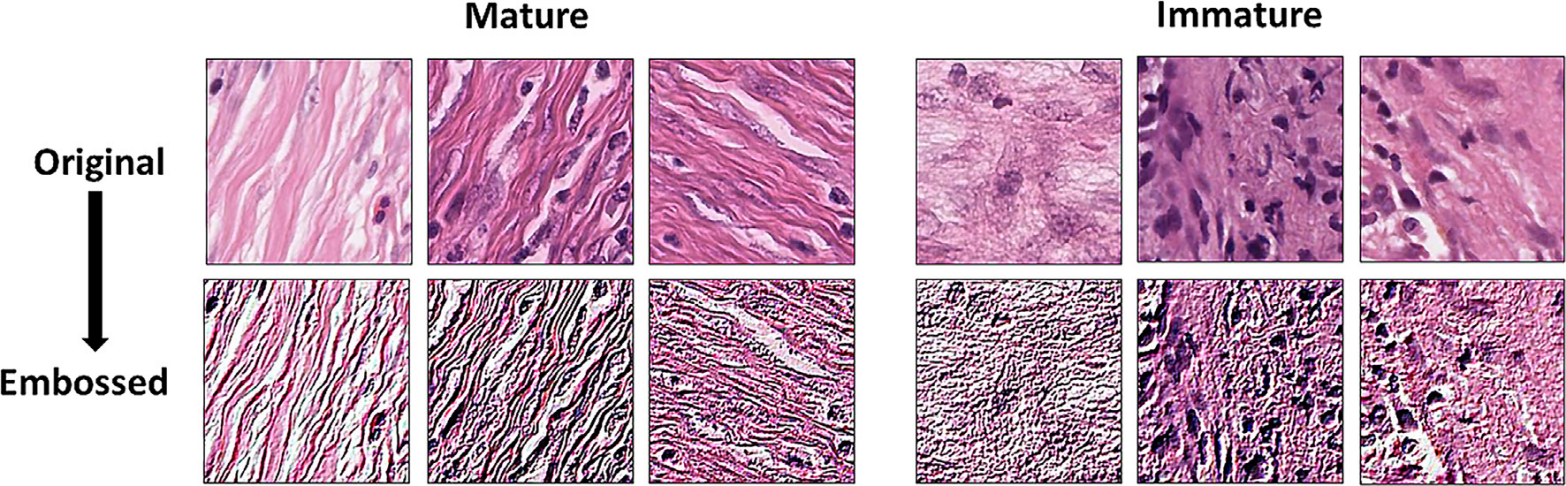
Demonstration of embossing on mature and immature tiles. The top row consists of the original images and the bottom row shows the effects of embossing. Embossing accentuates the difference between the two stroma maturities.

Class balancing augmented the smaller image class to provide the same number of images as the larger class. This option was chosen as reducing the larger class down, would have resulted in a lesser volume of images for training, harming model accuracy. Balancing the classes was done with the aim of reducing training bias towards a single class. Image pre-processing was limited to embossing of the images (Intensity = 2, Alpha = 1) (Fig. 6). Embossing was chosen with the aim of accentuating the differences in the features between mature and immature stroma outlined in Section 2.3. The same target image was used in all normalised sets. Normalisation was done with the aim of removing any potential colour bias in the model. In particular, the histogram matching technique was chosen here as it offered less computational overheads than other more advanced stain normalisation methods such as the Reinhard [44] and Macenko [45] methods, with the understanding that this may cause image artefacts [18]. All image manipulation was conducted prior to input in the CNN. The processes for creating all these image sets were timed. Augmentations were applied across the training, test and independent validation sets, with the exception of balancing, which was done across training and test sets only. HistoClean offers the ability to save to any servers connected to the computer operating system. As such these separate image sets were saved to a local server

### 2.6 CNN design

**Fig. 7:**
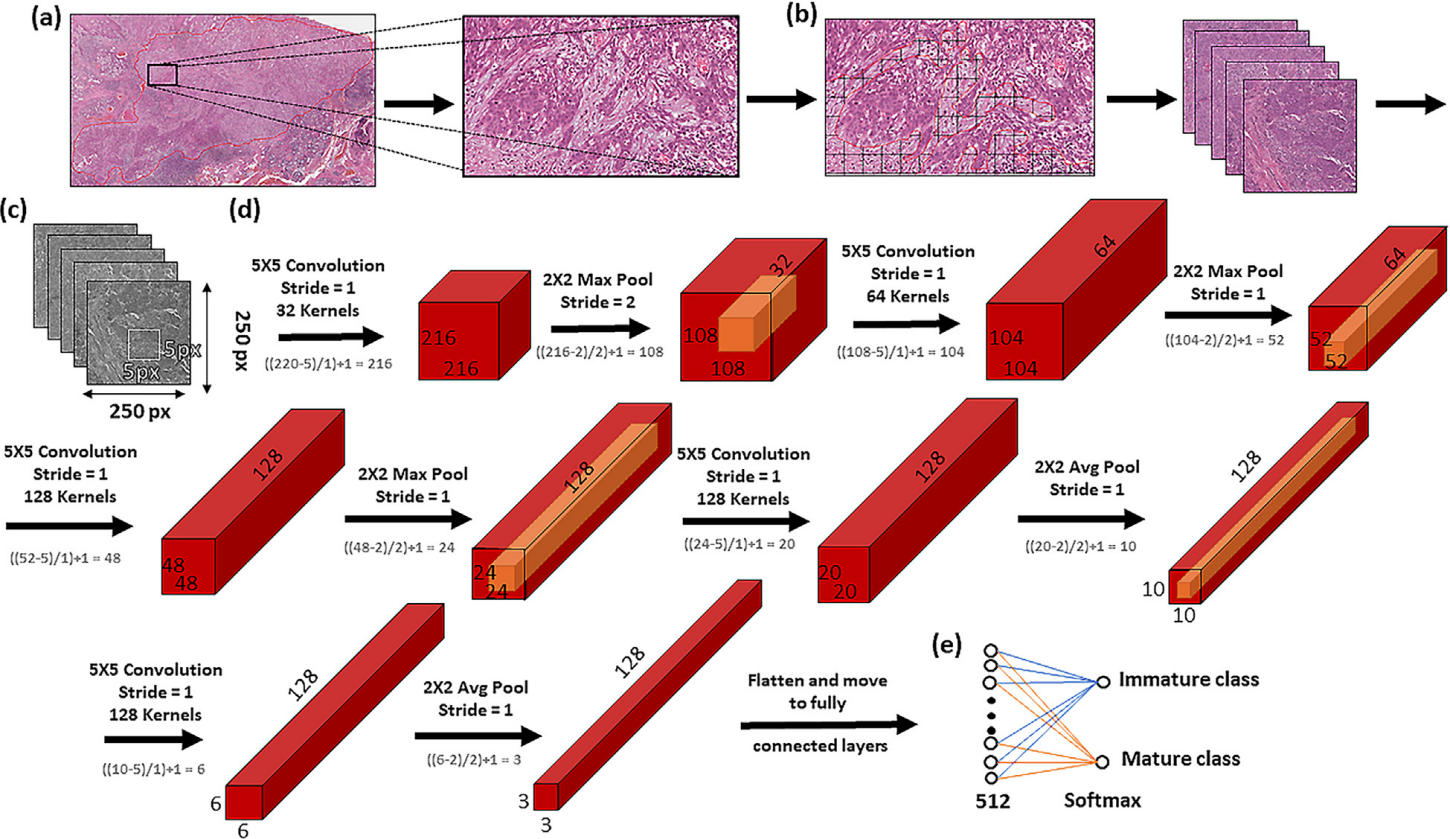
Workflow and architecture for the in-house convolutional neural network (CNN) used in the study. Regions of interest (ROI) are annotated and extracted from the tumour body (a). Image tiles of size 250x250 pixels were extracted from within stroma annotations within each ROI (b). Tiles were converted to greyscale to conserve memory, and fed into a CNN consisting of five convolutional layers interlinked with five pooling layers (c). A graphical representation of how these tiles are then processed within the five convolutional layers interlinked with five pooling layers of the CNN used in this study (d);the output of which is flattened before being fed into two fully connected layers wherein stromal maturity is predicted using the softmax function in the final layer (e). *(Avg = Average, Max = Maximum) Equations in grey show how feature map dimensions were calculated.*

The CNNs used in these experiments were designed using PyTorch [46]. A core CNN architecture was established and trained independently on each of the 8 datasets from scratch. This network consists of five convolutional layers interlinked with five pooling layers (Fig. 7). The output of the final pooling layer is then flattened and fed into two fully connected layers wherein stromal maturity is predicted using the softmax function in the final layer. The CNN architecture was kept relatively simple to reduce computational cost and training times, as well as highlight the impact of image pre-processing using HistoClean. Training was carried out for 200 epochs, with a batch size of 150. Adam Optimisation was used with a learning rate of 1e-6. Test batch size was set to 150 images. The outcome of the softmax function in the CNN produced a probability for each input image ranging from 0 (predicted mature) to 1 (predicted immature). Stromal maturity of the input images was classified as immature if the stromal maturity probability was greater or equal to 0.5, otherwise it was considered mature. After training on every fifth batch, the neural network calculated the accuracy and loss on a randomly selected test batch. If the test accuracy was greater than or equal to 65%, the weights and biases of the model were saved for further model evaluation. The weights and biases of the top 10 test batch accuracies were applied to the entire test set to get an improved evaluation of in-model performance. Only the model weights and biases that provided the top test accuracy were carried forward. These were then loaded to the CNN and applied to the independent validation image set. Stromal maturity probabilities at a ROI level were produced by majority voting of individual tile classifications. In patients with heterogeneous ROI classification of stromal maturity, majority voting of the ROIs was used to determine classification at a patient level. This was done to remain comparable with manual assessment. If the number of predicted stromal immature and mature ROI’s was equal the patient was considered to have mature stroma overall. To enable comparison of how different input images affected training of the CNN, batch size, learning rate, loss function and optimiser were all kept constant through all experiments. Full code for the CNN can be found at: (https://github.com/HistoCleanQUB/HistoClean)

### 2.7 Statistical analysis

The pathologist stromal maturity scores were used as the ground truth for development of the CNN. Model evaluation was conducted against the ground truth (pathologist scores) for the best-saved weights and bias in each of the image data sets at an individual tile, ROI and patient level. Confusion matrices were calculated to help determine the model’s precision, recall and F1-scores. Receiver-Operator Characteristic (ROC) curves were generated for assessment of the area under the curve (AUC) using the Scikit-learn library [34] in Python 3.8 at a tile and ROI level. Due to the heterogenous nature of some of the patients and methods of aggregation to predict outcome, ROC curves were not generated at this level.

Comparability between the best CNN model and the manual evaluation method was also assessed. Sensitivity, specificity, accuracy and their 95% confidence intervals were also calculated in the two additional independent manual stromal maturity classifications. For the purpose of this analysis, the model was considered a fourth evaluator. Inter-evaluator concordance was conducted using Fleiss’ Kappa. All bio-statistical analyses were performed using R v3.6.1 [47].

## 3 Results

### 3.1 Patient images

**Fig. 8:**
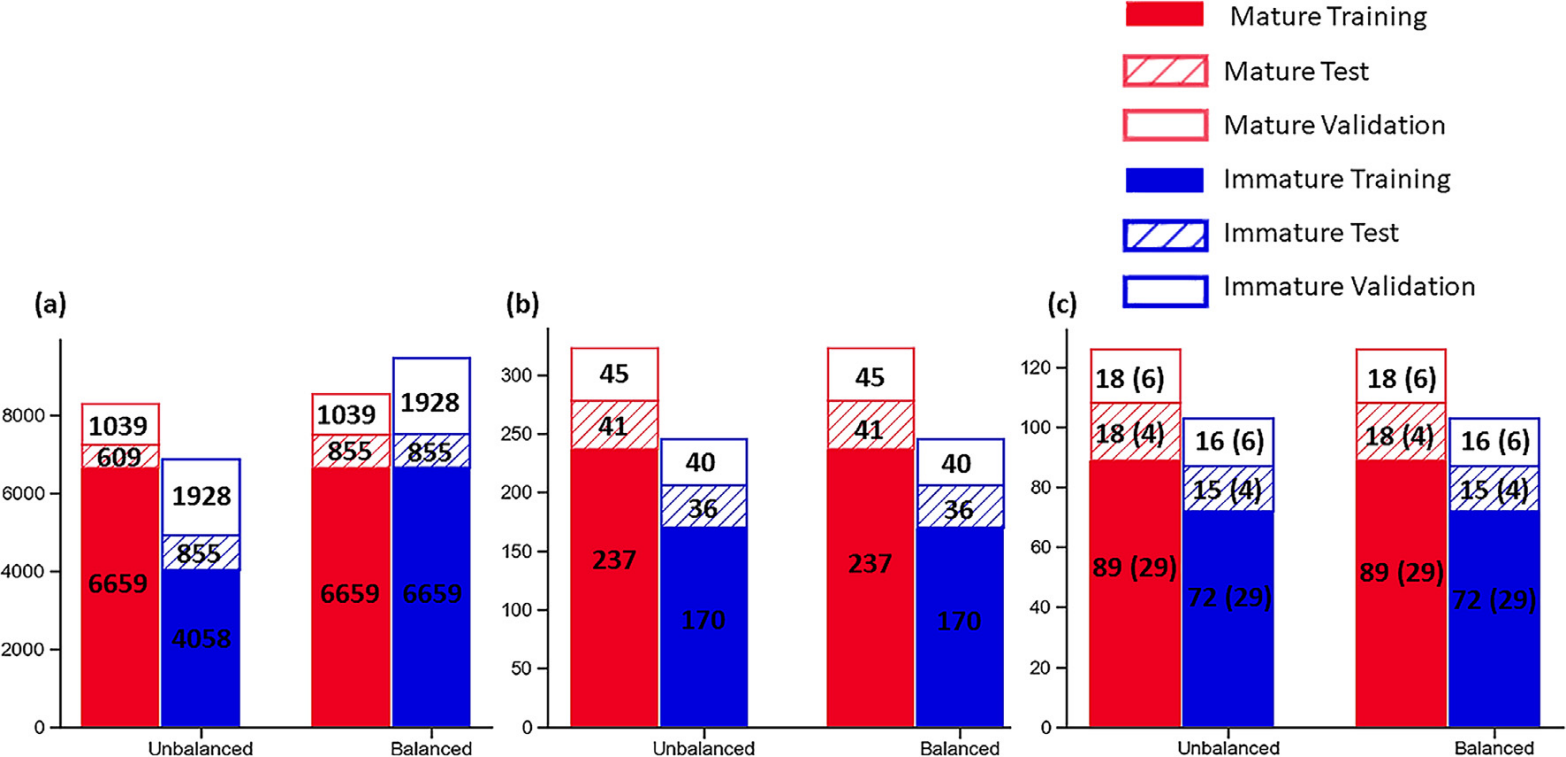
Histograms Showing breakdown of the image dataset for image tiles (A), ROIs (B) and at patient level (C) before and after dataset balancing. Datasets were only balanced at a tile level. NB The patient counts treat stromal heterogenous cases as both a mature an immature patient in these figures. The number of heterogenous patients are denoted in the parenthesis in (c).

Classification of stromal maturity in digitally annotated ROI’s was conducted on H&E stained slides for 197 patients with OPSCC. From these patients, 636 ROIs were annotated and evaluated manually. In total, 9.91% (63/636) ROIs had insufficient stroma to produce tiles, resulting in 4.06% (8/197) patients being excluded from further analysis in the study. Of the remaining patients, 33.86% (64/189) were found to have immature stroma in all ROIs assessed and 45.50% (86/189) patients were found to have mature stroma present in all ROIs assessed. Classification of stromal maturity across ROIs was heterogeneous in 20.64% (39/189) of patients assessed. There were 29 heterogenous patients in the training group, 4 in the test group and 6 in the independent validation group (Fig. 8). A complete breakdown of tiles, ROIs and patients can be found in Supplementary Figure 1.

### 3.2 Image set times

The time taken to perform each of the adjustments outlined in Table 1 were recorded for each image set. HistoClean balanced the baseline training data in 3.34 s with a difference of 2601 images translating to a rate of 778.74 images per second. Normalisation of all 15,148 images in unbalanced training data took 62.49 s equating to a rate of 242.40 images per second. Embossing the unbalanced data took 33.73 s, a rate of 449.10 images per second.

The use of multithreading allowed for the processing of the images in a rapid timeframe. As mentioned previously, the number of threads used scales with the CPU cores, allowing the user to carry out other tasks while HistoClean produces the new images.

### 3.3 Evaluation of image data sets in robust CNN development

**Fig. 9:**
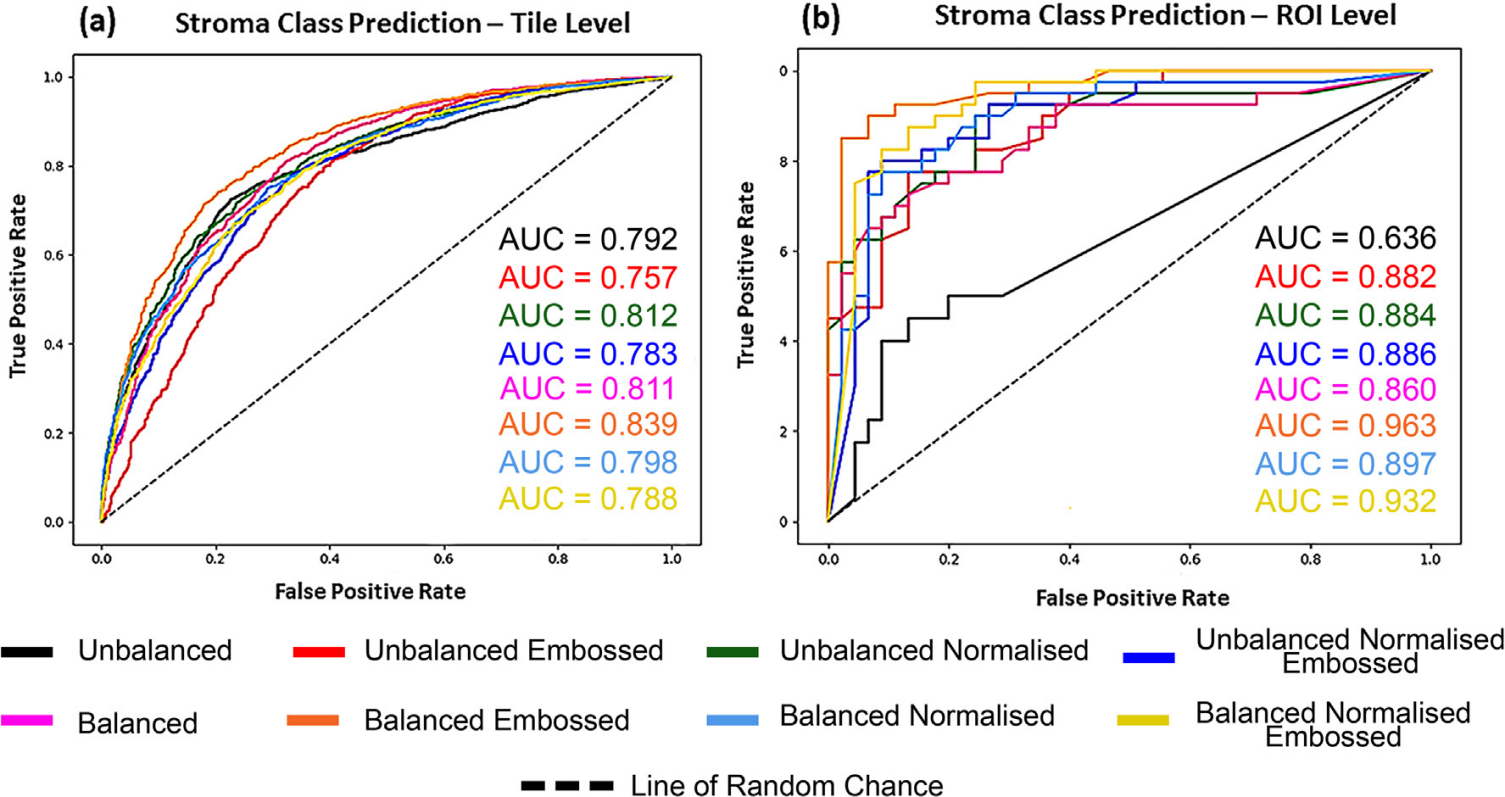
ROC Curve comparison of the different image datasets evaluated for CNN model accuracy within the image tiles (a) and ROIs (b). A combination of embossing and balancing the image sets provided the best overall area under curve (AUC) at a tile and ROI level.

The CNN was trained eight separate times from scratch using the eight separate image sets summarised in Table 1. Use of image pre-processing techniques were found to consistently improve upon model performance when compared to the baseline “unbalanced” dataset across all levels of prediction assessed; from probability of individual image tiles to aggregation of probability at the patient level (Table 2). Image pre-processing conducted in the Balanced Embossed set provided the best overall accuracy at a tile, ROI and Patient level (0.774, 0.835 and 0.857 respectively) as well as a superior f1-score (0.820, 0.844 and 0.846 respectively). From these results, the balanced embossed set was determined to be the best preforming image set overall. In addition, the Balanced Embossed image set provided the best area under curve (AUC) scores (0.839 and 0.963 at a tile and patch level; Fig. 9).

**Table 2:** Breakdown of model evaluation for each image set. Highlighted in bold are the best results for each category.

Image Set	Level	True Mature (TN)	False Immature (FP)	True Immature (TP)	False Mature (FN)	Precision	Recall	F1 Score	Total Correct	Total Incorrect	ROC AUC	Overall Accuracy
Unbalanced	Tile	961	78	746	1182	**0.905**	0.387	0.542	1707	1260	0.791	0.575
	ROI	41	4	12	28	0.75	0.3	0.429	53	32	0.636	0.624
	Patient	14	1	3	10	0.75	0.231	0.353	17	11	NA	0.607
Unbalanced Embossed	Tile	733	306	1281	647	0.807	0.664	0.729	2014	953	0.757	0.679
	ROI	29	16	34	6	0.68	0.85	0.756	63	22	0.882	0.741
	Patient	10	5	10	3	0.667	0.769	0.714	20	8	NA	0.714
Unbalanced Normalised	Tile	817	222	1314	614	0.855	0.682	0.759	2131	836	0.811	0.718
	ROI	34	11	35	5	0.761	0.875	0.814	69	16	0.884	0.812
	Patient	11	4	11	2	0.786	0.769	0.777	20	8	NA	0.714
Unbalanced Normalised Embossed	Tile	772	267	1316	612	0.831	0.683	0.75	2088	879	0.782	0.704
	ROI	33	12	37	3	0.755	0.925	0.831	70	15	0.886	0.824
	Patient	12	3	11	2	0.786	0.846	0.815	23	5	NA	0.821
Balanced	Tile	847	192	1216	712	0.864	0.631	0.729	2063	904	0.811	0.695
	ROI	37	8	30	10	**0.789**	0.75	0.769	67	18	0.86	0.788
	Patient	14	1	9	4	**0.9**	0.692	0.783	24	5	NA	0.828
Balanced Embossed	Tile	764	275	1532	396	0.848	0.795	**0.82**	2296	671	**0.839**	**0.774**
	ROI	33	12	38	2	0.76	0.95	**0.844**	71	14	**0.963**	**0.835**
	Patient	13	2	11	2	0.846	0.846	**0.846**	24	4	NA	**0.857**
Balanced Normalised	Tile	742	297	1422	506	0.827	0.738	0.78	2164	803	0.798	0.729
	ROI	32	13	36	4	0.735	0.9	0.809	68	17	0.897	0.8
	Patient	11	4	11	2	0.733	0.846	0.786	22	6	NA	0.786
Balanced Normalised Embossed	Tile	623	416	1590	338	0.793	**0.825**	0.808	2213	754	0.788	0.746
	ROI	27	18	39	1	0.684	**0.975**	0.804	66	19	0.932	0.776
	Patient	9	6	13	0	0.684	**1**	0.813	22	6	NA	0.786

**Fig. 10:**
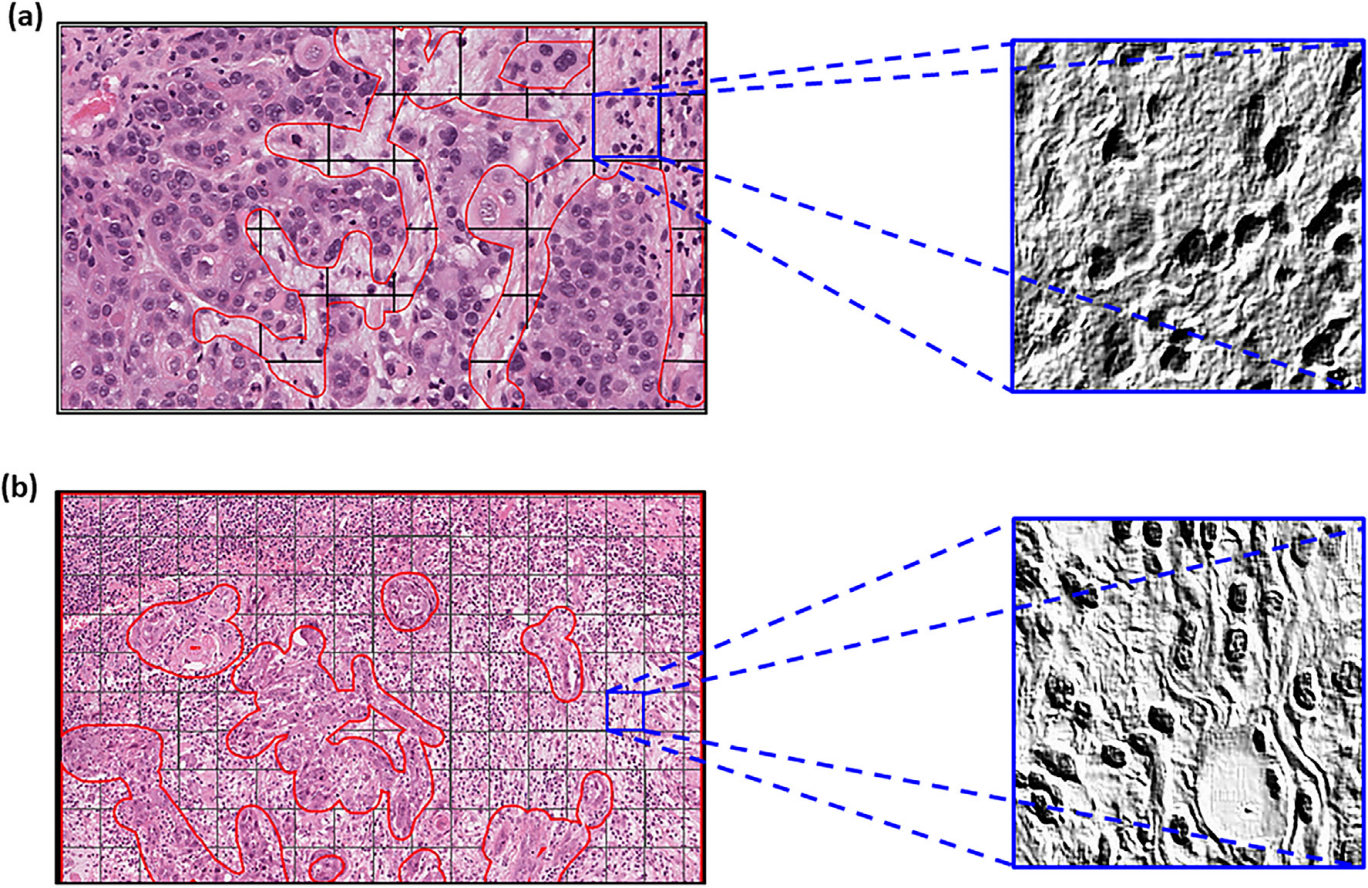
Representative examples of misclassified DS6 CNN stromal maturity prediction. Some patients in the cohort had limited stroma present, meaning very few tiles representative of overall patient’s stromal maturity could be extracted resulting in misclassification at a stromal independent patient level (a). Whilst at the tile level, image augmentation using the emboss technique was found to enhance linear structures surrounding oedema resulting in the embossed image possessing features associated with mature stroma resulting in misclassification of the tile (b).

The ability to predict stromal maturity using the CNN trained on the balanced embossed images was developed using the ground truth for stromal maturity in that ROI as provided by a single pathologist. Therefore, the sensitivity and specificity of manual classification of stromal maturity by two independent assessors to predict the pathologist scores was conducted and compared to results from balanced embossed image trained CNN in order to determine how reproducible the original pathologist scores were. Both independent manual assessors and the balanced embossed image set trained CNN demonstrated comparable sensitivity (100%; 95% CI, 77%–100%, for Assessor 1; 93%; 95% CI, 68%–100%, for Assessor 2 and 80%; 95% CI, 52%–96%, for the CNN) and specificity (86%; 95% CI, 57%–98%, for Assessor 1; 100%; 95% CI, 75%–100%, for Assessor 2 and 85%; 95% CI, 55%–98%, for the CNN) when classifying patients with having immature stroma based on the original pathologist scores. Moreover, the Fleiss’ Kappa score demonstrated good concordance between all three manual assessors and the CNN(κ = 0.785, p < 0.0001). A review of misclassification by the balanced embossed image set trained CNN found misclassification occurred most often when a small number of tiles were available for stromal classification in that patient (Fig. 10a). Misclassification by this model was found at a tile level whenever the image augmentation enhanced the presence of whitespace in immature stroma tiles resulting in misclassification of mature stroma in the embossed image (Fig. 10b). In one patient, no tiles were able to be extracted from 3 of the 5 ROIs, resulting in an inversion of stromal maturity prediction that was subsequently incorrect.

## 4 Discussion

As technology advances, so too does the demand for computational, high-throughput, cost-effective diagnostic tools for use in clinical medicine. This is particularly true in the field of clinical pathology that traditionally has utilised fewer technological aids in spite of a depleting workforce [48], [49]. Digital pathology, involves the acquisition and review of ultra-high-resolution whole slide images using a computer monitor in place of a microscope [50]. Digitisation of histological slides benefits from remote access for diagnostic reporting, providing a quick and easy means of recourse for diagnoses of complex pathology though ease of sharing virtual slides to consultant histopathologists with sub-specialist interest [48]. In addition, slide digitisation permits the use of digital image analysis tools to quantify histological features objectively using AI, as seen in radiomics [52]. At present, use of digital image analysis algorithms by consultant histopathologists is limited due to lack of modernisation in clinical pathology within the National Health Service, UK [51]. However, many consultant histopathologists recognise the benefit digital image analysis methodology could provide in streamlining the decision making process [53].

In contrast to other medical and non-medical disciplines that have implemented AI-assisted DIA, there is a scarcity of appropriate pathological images for developing deep learning models in clinical pathology [54]. This is in part due to the relatively recent move towards digitisation of pathology services, but more often due to lack of pathological material regarding the question of interest. Histological images are data rich and demonstrate significant heterogeneity across and within disease pathologies [55]. Therefore, the number of images required for effective deep learning is that of many orders of magnitude greater than that those required when developing models using more classical machine learning methods. Depending on the model being developed, this may require image datasets to be sourced at a global scale. Consequently, this introduces image variability and potential bias into CNN learning through differences in laboratory practice, scanning procedures or age of the sample being scanned [56]. This can have a pronounced effect on model learning and validation, particularly in small cohort studies, as each histological image possess unique image colour, contrasts and brightness profiles. The inter-laboratory variation limits the efficacy of developed models from small cohort students to be used in practice. CNNs have already shown promise in several cancer types and in several different use cases. One study by Khosravi et al. evaluated both in-house and the current top pretrained models’ efficacy across numerous cancer types and in several different tasks [57]. Many of these models achieved > 90% accuracy in the categories of tumour detection, biomarker detection and tumour subtyping in bladder, breast and lung cancers. Another study demonstrated the use of several pretrained neural networks to identify different growth patterns in lung adenocarcinoma, achieving accuracies up to 85% [6].

In this study, we demonstrate the power of image pre-processing and augmentation and present a novel open-source GUI called HistoClean. Using a relatively simple CNN architecture, we clearly establish how use of image pre-processing techniques improves upon model generalisability for prediction of stromal maturity in an independent validation dataset. Further, we show that the best developed model, the balanced embossed model, had similar concordance, sensitivity and specificity to two further independent assessors of stromal maturity by manual review. However, we also show that poor choice of image pre-processing and augmentation techniques can introduce bias and noise. The use of image augmentation for dataset balancing helped to increase the small number of immature samples present for model development whilst image pre-processing through embossing helped to accentuate the features of interest we wanted the model to train with. Therefore, to ensure successful model development, consideration of which techniques to implement should reflect the specific research question being asked. HistoClean offers a simple point-and-click GUI that allows users without a coding background to rapidly augment and pre-process images, utilising live feedback to evaluate these changes. This also aids computer scientists by removing the process of writing, running and re-running scripts. The minimalistic user interface, combined with the provided procedural instruction, creates an implicit user-friendly experience [22], [23], [25].

When trying to improve the accuracy of a CNN, often developmental time is spent refining the neural network and the network’s hyperparameters, or using deeper networks. However, it is arguably just as, if not more important to focus on the quality of the images used in training the network; a sentiment captured by the expression “rubbish in = rubbish out”. This study illustrates how crucial it is to balance the number of input images across the classes to prevent model overfitting. This initial step significantly improved both overall accuracy and AUC at the tile, patch and ROI level. The strength of this action is also clearly demonstrated by the change in false mature and false immature rates when comparing the balanced dataset to the unbalanced dataset. This is evidenced in the increases in f1-value at tile ROI and patient level (0.187, 0.340 and 0.443 respectively, Table 2). In parallel to this, embossing alone also demonstrated increases in accuracy and AUC across all levels, as well as lessening the effect of a mature dominant training set (Table 2). A synergistic improvement occurred when the dataset was both balanced and embossed, achieving an accuracy of 0.774 at a tile level. These improvements are in line with several other studies that use different augmentation techniques [58], [59], [60]. Importantly, HistoClean allowed the bio-image analyst to review the output of the image processing steps being applied within the software before proceeding to model development, providing opportunity for discussion of how particular image augmentations may enhance qualitative features the pathologist used to define stromal maturity in the image.

The CNN used in this study is relatively simple. This case-study demonstrates that high quality input data for training through the use of both pre-processing and augmentation techniques can improve classification accuracy with simple model architecture. Future studies utilising these same image augmentation and pre-processing techniques for more advanced deep learning models such as VGG [61], AlexNet [62]and ResNet [63] architectures would be of interest. The positive impact of these techniques may be less pronounced in these models due to the higher complexity of the models. However, this would be at a much greater computational cost and training times, as well as requiring more high-powered computer hardware that creates a barrier to entry for deep learning.

While HistoClean has proven to be a useful tool in this study, there are improvements which can be made. At this current time, HistoClean uses the CPU only to process images, which may somewhat limit the operation speed of the application. The tasks carried out here could well benefit from GPU integration in future releases. It is also important to note that HistoClean has to date only been tested utilising H&E-stained images and further development is required for immunohistochemistry-based applications. In addition, an application like this may benefit from direct integration into Python and PyTorch, so the addition of a function to export the augmentations as a Python script may be valuable and improve reproducibility. However, in its current state HistoClean can still help inform the augmentation techniques used at runtime. Future versions of HistoClean could be developed to create runtime-based image augmentation scripts in conjunction with data loaders, avoiding the requirement of saving the newly created images directly to disc. Finally, at present, the user is required to have already produced the tiles from the whole slide images before using HistoClean. The application would benefit from the introduction of a slide viewer and annotation tool for ROI-specific tile extraction to truly be an all-in-one toolbox. The version of the Image Patching module in this study only accepts .png and .jpg files, and would avail from compatibility with proprietary whole slide image formats such as .svs and .ndpi files, which could be introduced in future versions through the OpenSlide library [2]. Furthermore, the ability to explicitly select the output magnification of the tiles from these images directly from HistoClean would be desirable. The open-source nature of this software provides the possibility for community driven growth and development. This, in combination with continued support of the creators, will allow HistoClean to continue to grow and add more complex techniques in the future.

**Fig. 11:**
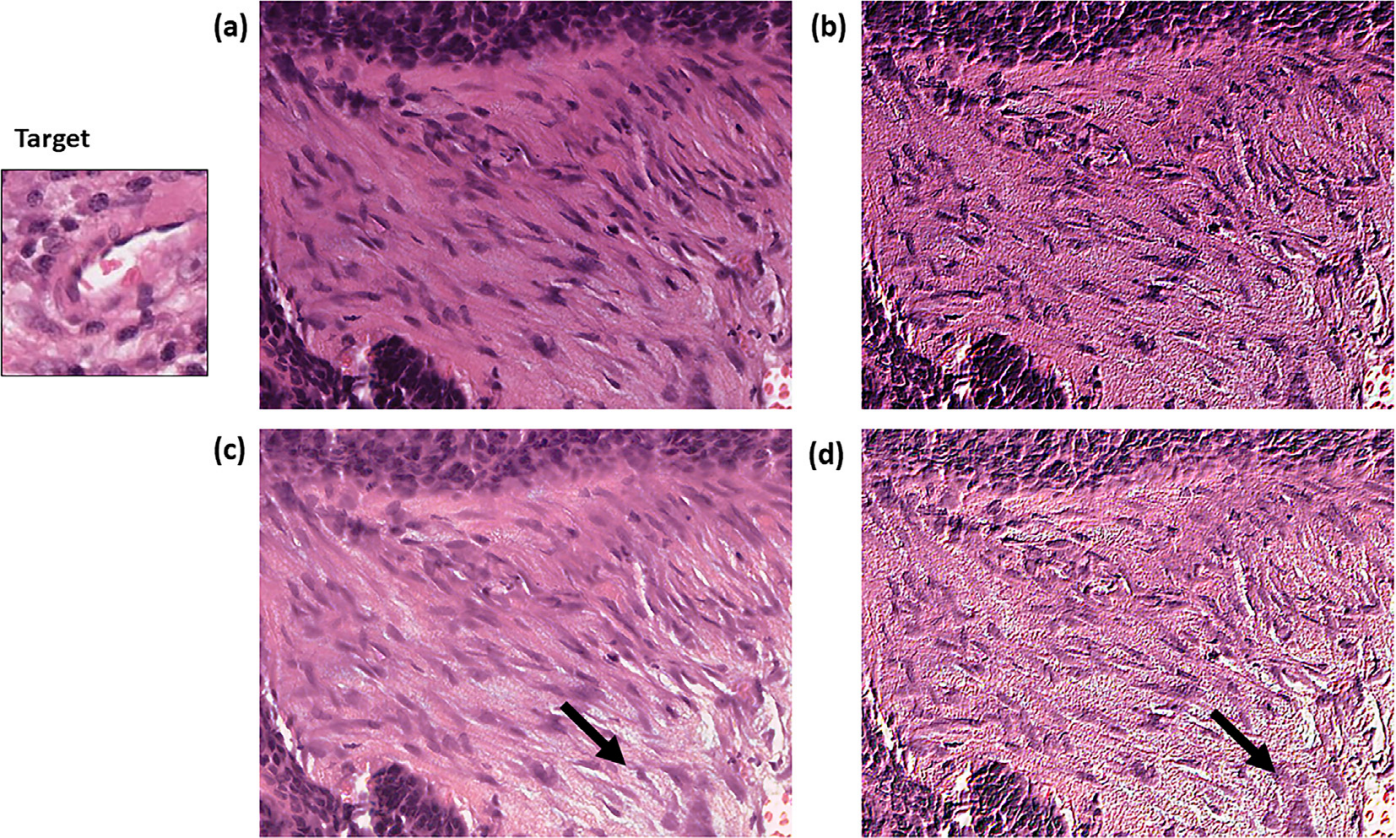
Example ground truth mature ROI. The original image (a) was embossed (b) and in the balanced embossed image set was predicted mature. Normalisation of the image created artefactual whitespace (c) which was then exacerbated by the embossing (d), flipping the prediction to an immature phenotype.

In this study, we also demonstrate that inappropriate augmentations can harm deep learning model development. This is evidenced by the reduction in accuracy between the Balanced Embossed and Balanced Normalised Embossed image sets, with a particular shift towards immature prediction as reflected in the increase in recall and decrease in precision at all levels. Upon examination of the patients in which this phenomenon had the greatest effect, it was clear that image normalisation, while correcting any colour imbalance, often created artefactual whitespace (Fig. 11c). This was further highlighted by the embossing, (Fig. 11d) causing the mature tiles to lose the dense parallel stromal fibres and adopt a more immature phenotype. This also raises the question of whether the improvements between the unbalanced and unbalanced normalised image sets are genuine or an artificial correction in the majority mature training data. It could be hypothesised that an immature skewed training set could suffer from further negative bias using this technique. Situations like this reinforce HistoClean as a useful tool for image pre-processing. A trained pathologist would be able to preview these changes and identify flaws in the pre-processing steps to avoid them. Furthermore, the traceability and data integrity provided by the application allows for easy comparison of the images.

While the findings of this work give reason to be optimistic, there are still barriers to overcome before these tools are utilised in a clinical setting. With the common complaints of job losses and disconnect from the patient [64] aside, there can also be a lack of explainability and interpretability of the outcomes of neural networks; known as “Black Box” Deep learning [65]. This has led to a debate on how important it is to explain diagnostic outcome even if the accuracy is high [66]. However, this is comparable to the many commonly used drugs where we still lack a complete understanding of their mechanism of action [67]. There have been great efforts made to help uncover the logic behind image classification in deep learning models. These include the generation of saliency maps based on the generated gradients and loss [68], gradient-weighted class activation mapping [69], and minimal explainability maps ([70]). These techniques highlight areas of interest on the original images, providing some insight into which features are contributing to the classification. As techniques like this continue to improve, the concerns around the blind nature of deep learning should be alleviated.

## 5 Conclusions

This study confirms that use of image pre-processing and augmentation techniques available in HistoClean can advance the field of deep learning by facilitating arguably the most important step CNN-centric experiments; image set preparation. However, there is a lack of easy to use open-source GUI software to facilitate this process, and therefore this often requires knowledge of computer programming. This study demonstrates the usefulness of HistoClean as an open-source software to implement image pre-processing techniques in image research, saving time and improving transparency and data integrity. HistoClean provides a rapid, robust and reproducible means of implementing these techniques in a way that can be used by experts, such as pathologists, to help identify which techniques could potentially be of use in their study, without the need for an inherent knowledge of coding. HistoClean also saves the user the effort of running and re-running scripts to assess how the pre-processing techniques may be affecting the underlying biology in the image. This in turn empowers the researchers by allowing them to better make judgements on the optimal techniques to apply for their work. The application has been designed around the concept of minimalism and procedural instruction to create an inherently user-friendly experience. The open-source nature of HistoClean allows for the continuous development of the application as more advanced augmentation and pre-processing techniques are identified and requested.

## CRediT authorship contribution statement

**Kris D. McCombe:** Conceptualization, Data curation, Software, Formal analysis, Validation, Investigation, Visualization, Methodology, Writing – original draft, Project administration, Writing - review & editing. **Stephanie G. Craig:** Conceptualization, Data curation, Formal analysis, Supervision, Validation, Investigation, Methodology, Writing – original draft, Project administration, Writing - review & editing. **Amélie Viratham Pulsawatdi:** Data curation, Investigation, Writing - review & editing. **Javier I. Quezada-Marín:** Data curation, Investigation, Writing - review & editing. **Matthew Hagan:** Data curation, Investigation, Writing - review & editing. **Simon Rajendran:** Data curation, Investigation. **Matthew P. Humphries:** Resources, Data curation. **Victoria Bingham:** Resources. **Manuel Salto-Tellez:** Resources, Funding acquisition, Project administration. **Richard Gault:** Conceptualization, Software, Supervision, Validation, Methodology, Writing – original draft, Project administration. **Jacqueline A. James:** Conceptualization, Resources, Data curation, Supervision, Funding acquisition, Methodology, Project administration.

## Declaration of Competing Interest

Dr. M.S.T has recently received honoraria for advisory work in relation to the following companies: Incyte, MindPeak, QuanPathDerivatives and MSD. He is part of academia-industry consortia supported by the UK government (Innovate UK). Dr J.J. is also involved in an academic-industry research programme funded by IUK. These declarations of interest are all unrelated with the submitted publication. All other authors declare no competing interests.

## Figures and Tables

**Supplementary figure 1:**